# Aberrant CD4 Expression in Plasma Cell Myeloma With Unusual Morphology: A Rare Diagnostic Pitfall

**DOI:** 10.7759/cureus.109182

**Published:** 2026-05-19

**Authors:** Gayathry Rajasekharan, Sunitha Balakrishnan, Resmi Rajeev

**Affiliations:** 1 Pathology, Government Medical College, Thrissur, Thrissur, IND

**Keywords:** cd4, extramedullary plasmacytoma (emp), kappa immunoglobulin light chain, multiple myeloma, plasma cell myeloma, plasmacytoma, t cell differentiation antigens

## Abstract

Plasma cell myeloma (PCM) is a clonal plasma cell neoplasm diagnosed by its characteristic morphology and immunophenotype. Rarely, it shows significant morphological and immunophenotypic heterogeneity, mimicking other hematolymphoid malignancies. This case highlights PCM with deceptive morphology and aberrant T-cell marker expression, underscoring a key diagnostic pitfall. A 62-year-old female presented with an indurated lesion on the left lateral aspect of the tongue and left shoulder swelling for one month, with associated constitutional symptoms. Laboratory investigations showed anemia, raised lactate dehydrogenase, serum creatinine, and serum calcium levels. Biopsy from both sites revealed a neoplasm composed of small- to medium-sized cells in diffuse sheets with mild nuclear pleomorphism. Immunohistochemistry done on the tongue lesion revealed positivity for CD138, CD38, light chain kappa, and CD4, with a Ki-67 index of 90%. A similar immunohistochemical profile was identified in the shoulder lesion. Both lesions were diagnosed as plasmacytoma, prompting evaluation for PCM. Imaging demonstrated an enhancing soft tissue density on the anterior half of the tongue, multiple sclerotic and lytic lesions, multiple metastatic lymph nodes, and multiple nodular deposits in the cervical region. Subsequent bone marrow examination revealed 90% marrow plasmacytosis with kappa light chain restriction, confirmed by immunohistochemistry. This concluded in a final diagnosis of PCM with kappa light chain restriction and aberrant CD4 positivity. The diagnosis was corroborated with further myeloma workup, which revealed an M band in serum electrophoresis, markedly elevated serum kappa free light chain, kappa:lambda ratio, and serum IgG. Diagnosis of PCM becomes challenging in atypical presentations, lacking classical morphology and immunophenotype, necessitating a comprehensive immunohistochemical panel and close clinicopathological correlation while accounting for lineage infidelity in poorly differentiated hematologic neoplasms to avoid diagnostic pitfalls.

## Introduction

Plasma cell neoplasms (PCNs) are clonal proliferations of plasma cells derived from terminally differentiated B-cells with varied clinical manifestations, most of which are linked to the production of a monoclonal immunoglobulin, or M protein, that is detectable in the serum and/or urine. According to the 2022 World Health Organization Classification of Hematolymphoid Tumors, PCNs are categorized as monoclonal gammopathies, light or heavy chain diseases, mass-forming plasmacytoma, plasma cell myeloma (PCM), or plasma cell leukemia with their corresponding paraneoplastic syndromes based on clinical manifestations and laboratory results [1].

Diagnosis of PCM requires either biopsy-proven bony or extramedullary plasmacytoma or >10% clonal bone marrow plasma cells along with one or more myeloma-defining events (MDEs) as per the International Myeloma Working Group criteria. MDEs comprise CRAB features (hypercalcemia: serum calcium >11 mg/dL; renal insufficiency: creatinine clearance <40 mL/min or serum creatinine >2 mg/dL; anemia: hemoglobin <10 g/dL; bone lesions: more than one osteolytic lesion on imaging) or biomarkers (greater than 60% clonal plasma cells; involved: uninvolved serum free light chain ratio >100 with involved level >100 mg/L; more than one focal lesion on MRI) [2].

The most common presentation is multifocal bone marrow involvement, lytic lesions, and tumor masses, especially at sites of active hematopoiesis. Besides, extramedullary involvement and the presence of circulating plasma cells are features pointing toward advanced disease [1]. Morphologically, plasma cells typically exhibit eccentric nuclei, clock-face chromatin, and eosinophilic cytoplasm on H&E-stained slides. The diagnosis is further supported by immunohistochemistry or flow cytometry demonstrating plasma cell markers, including CD38, CD138, and CD79a, with clonality confirmed by kappa or lambda light-chain restriction. In typical cases, this does not pose a diagnostic challenge. Diverse morphological and phenotypic presentations, as well as less frequently encountered unique immunophenotypic profiles, are well-known characteristics of PCNs. Such unanticipated immunophenotypic diversity may lead to false impressions during primary evaluation, delaying a precise diagnosis [3]. Thus, it often makes it challenging to differentiate PCM from other PCNs and other lymphoma types if they have unusual clinical, morphologic, and phenotypic characteristics [4]. Compounding this challenge, aberrant expression of T-cell markers like CD4 on neoplastic plasma cells is exceptionally uncommon, with only sporadic case reports describing such immunophenotypic anomalies [5,6]. Aberrant T-cell marker expression in a high-grade neoplasm lacking typical morphologic features of PCNs can lead to diagnostic confusion with entities such as peripheral T-cell lymphoma, not otherwise specified, extranodal NK/T-cell lymphoma, and plasmablastic lymphoma, thereby underscoring the importance of including plasma cell markers (CD138 and CD38) in the immunohistochemical panel even in the absence of overt plasmacytoid or plasmablastic morphology [7].

This report details a rare instance of PCM exhibiting aberrant CD4 expression alongside unusual morphology at presentation, highlighting diagnostic pitfalls and advocating for comprehensive multimodal assessment to guide precise classification and management.

## Case presentation

A 62-year-old female presented to the outpatient department with fatigue and left-sided shoulder swelling of one and a half months’ duration. Similar swellings were also noticed in the back, left arm, forearm, and undersurface of the tongue. This was associated with on-and-off fever, vomiting, loss of appetite, and generalized pain of the same duration. On local examination, a left-sided shoulder swelling and an indurated ulcerative lesion on the left lateral aspect of the tongue were identified. Initial blood investigations identified anemia, raised erythrocyte sedimentation rate, increased serum creatinine, hyperglobulinemia, reversal of albumin/globulin (A/G) ratio, raised lactate dehydrogenase (LDH), and negative serological markers for hepatitis C virus, HIV, and HBsAg. The results of investigations are summarized in Table 1.

**Table 1:** Initial laboratory investigations Initial hematological and biochemical investigations showing anemia, raised ESR, increased serum creatinine, hyperglobulinemia, reversal of A/G ratio, and raised LDH. A/G, albumin/globulin; ESR, erythrocyte sedimentation rate; LDH, lactate dehydrogenase; SGOT, serum glutamic-oxaloacetic transaminase; SGPT, serum glutamate-pyruvate transaminase

Test	Result	Normal range
Hemoglobin	6.7 g/dL	12-15 g/dL
Total leukocyte count	8700/mm³	4000-10000/mm³
Neutrophil	56%	40-80%
Lymphocyte	42%	20-40%
Eosinophil	2%	1-6%
Platelet	1.5 lakh/mm³	1.5-4.1 lakh/mm³
ESR	80 mm/hour	<20 mm/hour
Serum creatinine	1.5 mg/dL	0.6-1.1 mg/dL
Total protein	7.7 g/dL	6.0-8.3 g/dL
Albumin	3.5 g/dL	3.5-5.0 g/dL
Globulin	4.2 g/dL	2.0-3.5 g/dL
A/G ratio	0.83	1.0-2.2
Serum calcium	10.2 mg/dL	8.5-10.5 mg/dL
SGOT	18 U/L	5-40 U/L
SGPT	15 U/L	7-56 U/L
LDH	525 IU/L	140-280 IU/L

Ultrasonography of the neck identified a large, well-defined, heterogeneous lesion, predominantly hypoechoic, with internal vascularity in the bilateral sternocleidomastoid muscles. CT neck and upper chest revealed soft tissue density in the anterior half of the tongue measuring 3.7 × 2.8 × 2.7 cm (Figure 1). Multiple large heterogeneous nodular deposits were noted in the right sternocleidomastoid, right subclavicular area, surrounding left shoulder joint, right masseter, bilateral pterygoid region, floor of mouth, right paravertebral region, along the right vertebral artery, left tonsil, right aryepiglottic fold, and right maxilla (Figure 2, Figure 3). Multiple enhancing nodular subcutaneous deposits were identified in the bilateral anterior chest wall and bilateral upper back. Bilateral cervical lymphadenopathy was present. Multiple sclerotic lytic bony lesions in the maxilla, mandible, cervical and thoracic vertebrae, ribs, base of skull, bilateral clavicles, and scapula were noted. All findings were suggestive of a malignant etiology.

**Figure 1:**
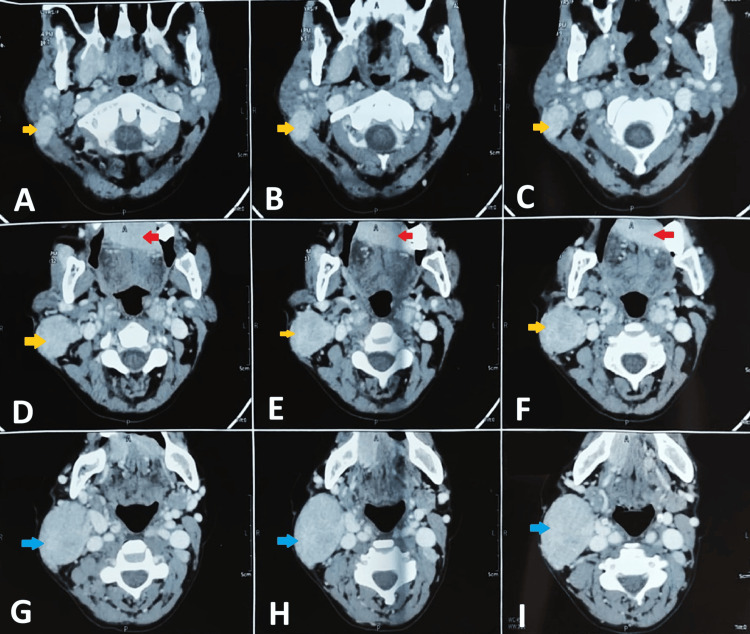
CT neck (plain and contrast) Heterogeneously enhancing soft tissue density in the anterior half of the tongue (red arrow in D-F) measuring 3.7 × 2.8 × 2.7 cm, with large nodular deposits in the right masseter (yellow arrow in A-F) measuring 1.4 × 1.2 cm, the right sternocleidomastoid (blue arrow in G-I), and the right paravertebral region along the right vertebral artery.

**Figure 2:**
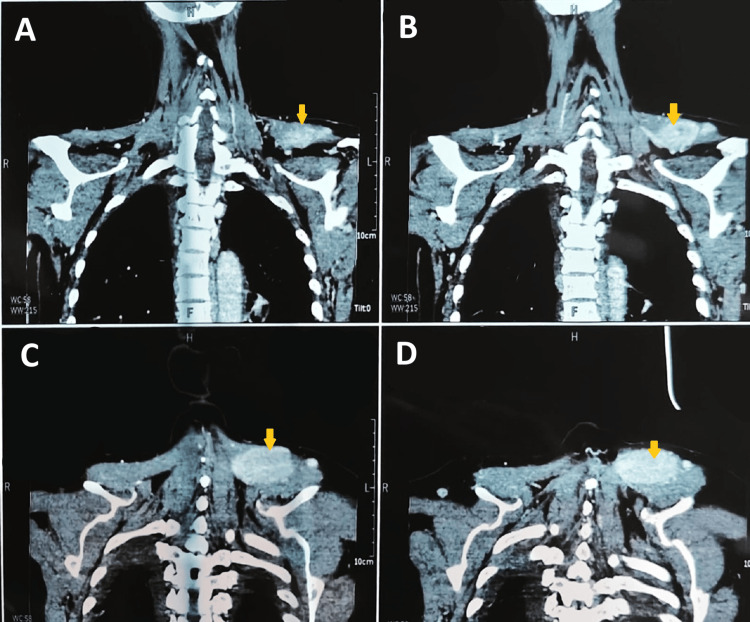
CT neck and chest (plain and contrast) (A-D) Large heterogeneously nodular deposits surrounding the left shoulder joint (yellow arrow) measuring 12 × 3.3 cm.

**Figure 3:**
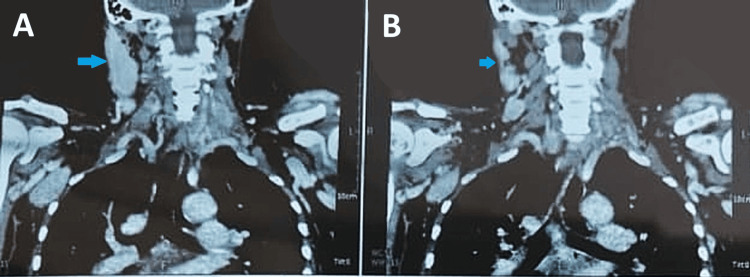
CT neck and chest (plain and contrast) (A, B) Large heterogeneously enhancing nodular deposit in the right sternocleidomastoid muscle (blue arrow) measuring 5.2 × 7 cm.

An incisional biopsy from the left lateral border of the tongue and a Tru-Cut biopsy of the left shoulder swelling were done. Histopathological examination of the tongue biopsy revealed an infiltrating neoplasm in the subepithelium, composed of small- to medium-sized cells arranged in diffuse sheets, cords, and trabeculae. The individual cells exhibited scant eosinophilic cytoplasm and pleomorphic vesicular nuclei, with some showing distinct nucleoli. Mitoses were observed at 1-2 per 10 high-power fields (Figure 4). A similar histomorphological picture was recognized in the shoulder biopsy as well (Figure 5). In both cases, the findings prompted consideration of different possibilities, particularly a neuroendocrine tumor and a lymphoproliferative neoplasm.

**Figure 4:**
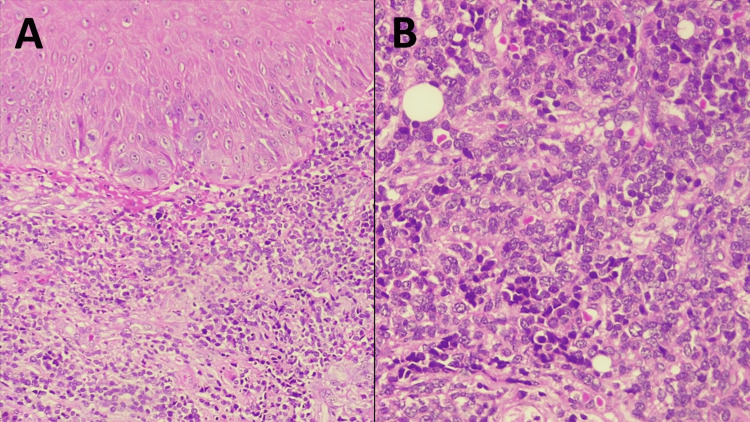
H&E images of the biopsy from the tongue (A) Subepithelium showing an infiltrating neoplasm composed of small- to medium-sized cells in diffuse sheets (100×). (B) Individual cells with scant eosinophilic cytoplasm and pleomorphic vesicular nuclei, with some showing distinct nucleoli (400×).

**Figure 5:**
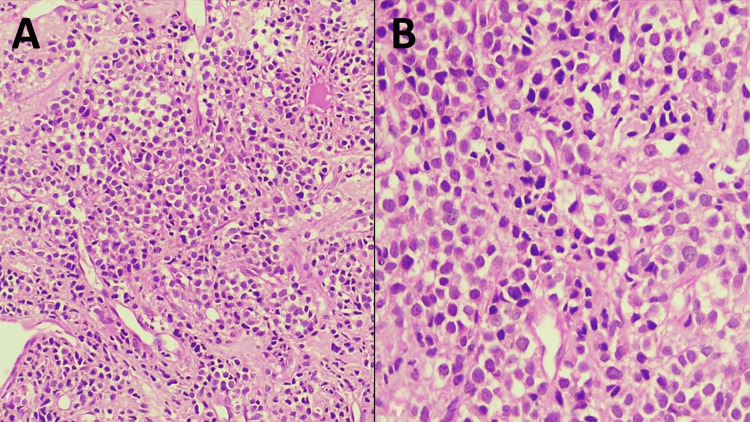
H&E images of the biopsy from the shoulder lesion (A) Infiltrating neoplasm in diffuse sheets and trabeculae (100×). (B) Individual cells with scant to moderate cytoplasm, mildly pleomorphic hyperchromatic nuclei (400×).

For the purpose of subcategorization of the neoplasm, immunohistochemical evaluation was done on the biopsy from the tongue lesion, which revealed strong positivity for CD138, CD38, light chain kappa, and CD4 in the neoplastic cells. These cells were negative for light chain lambda, CD56, CD5, CD20, CD10, TdT, CK, LCA, synaptophysin, chromogranin, CD23, INSM1, NSE, cyclin D1, and CD68. The Ki-67 index was approximately 90% in hotspot areas (Figure 6). Additionally, immunohistochemical analysis performed on the biopsy from the shoulder lesion revealed a similar profile (Figure 7). Correlating light microscopy, immunohistochemistry, and imaging, a diagnosis of plasmacytoma with aberrant CD4 positivity was given.

**Figure 6:**
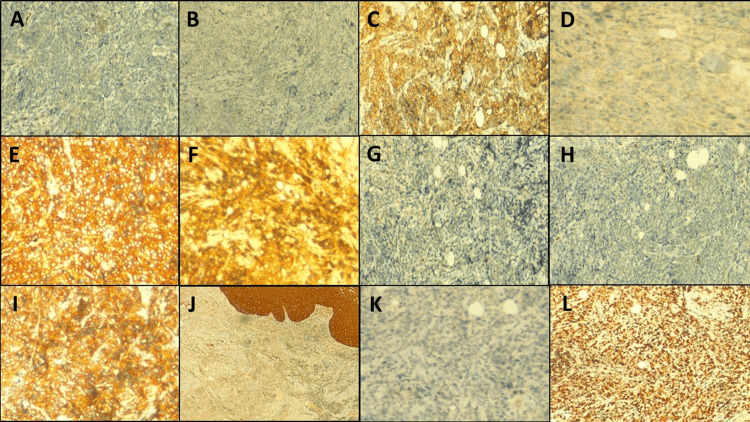
Immunohistochemical images of the biopsy from the tongue (A) CD5 negative. (B) CD20 negative. (C) CD38 diffuse moderate membranous positivity. (D) Light chain lambda negative. (E) CD138 diffuse, strong membranous positivity. (F) Light chain kappa shows strong diffuse cytoplasmic positivity. (G) CD68 negative. (H) Chromogranin negative. (I) CD4 diffuse, strong membranous positivity. (J) PanCK negative (positive internal control). (K) Insulinoma-associated protein 1 (INSM1) negative. (L) Ki-67 index 90% in hotspot areas.

**Figure 7:**
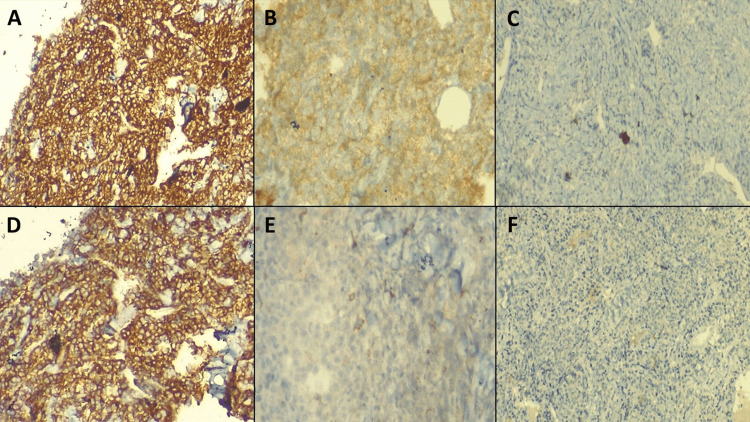
Immunohistochemical images of the biopsy from the shoulder lesion (A) CD138 diffuse, strong membranous positivity. (B) CD4 diffuse moderate membranous positivity. (C) Light chain lambda negative. (D) Light chain kappa shows diffuse, strong cytoplasmic positivity. (E) LCA negative. (F) Chromogranin negative.

On further follow-up after two weeks, a fall in hemoglobin level and a rise in serum creatinine, calcium, and LDH levels were observed (Table 2). The patient was subsequently managed symptomatically with packed red cell transfusions due to falling hemoglobin levels.

**Table 2:** Follow-up laboratory investigations, including myeloma workup Subsequent hematological and biochemical investigations showing a fall in hemoglobin and albumin levels; an increase in serum creatinine, globulin, serum calcium, and LDH levels; and a reversal of the A/G ratio. Myeloma workup revealed increased serum free light chain (kappa), raised serum free light chain ratio (>100), raised serum IgG, and serum beta-2 microglobulin. A/G, albumin/globulin; LDH, lactate dehydrogenase

Test	Result	Normal range
Hemoglobin	6.2 g/dL	12-15 g/dL
Serum creatinine	2.6 mg/dL	0.6-1.1 mg/dL
Total protein	7.7 g/dL	6.0-8.3 g/dL
Albumin	3.4 g/dL	3.5-5.0 g/dL
Globulin	4.30 g/dL	2.0-3.5 g/dL
A/G ratio	0.79	1.0-2.2
Serum calcium	11.3 mg/dL	8.5-10.5 mg/dL
Serum kappa free light chain	1191.53 mg/L	3.3-19.4 mg/L
Serum lambda free light chain	8.34 mg/L	5.7-26.3 mg/L
Serum kappa:lambda ratio	142.86	0.26-1.65
Serum IgG	2128 mg/dL	700-1600 mg/dL
Serum IgM	33 mg/dL	40-230 mg/dL
Serum IgA	35.277 mg/dL	70-400 mg/dL
LDH	827 IU/L	140-280 IU/L
Serum beta-2 microglobulin	7.2 mg/L	0.7-1.8 mg/L

In view of the biopsy findings, the patient underwent a bone marrow examination, which revealed 90% marrow plasmacytosis, with plasma cells arranged in sheets, nodules, and diffusely. Quantification of plasma cells was performed with immunohistochemistry for CD138, which also revealed light chain kappa restriction (Figure 8). 

**Figure 8:**
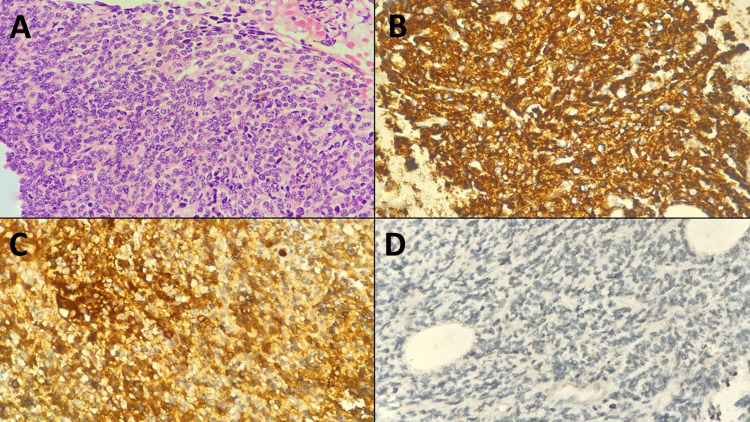
Images of bone marrow biopsy (A) Sheets of plasma cells with abundant cytoplasm, eccentric nuclei, and clock-face chromatin (H&E, 400×). (B) CD138 diffuse, strong membranous positivity (plasmacytosis 90%). (C) Light chain kappa shows diffuse, strong cytoplasmic positivity. (D) Light chain lambda negative.

Additional myeloma workup revealed an M band on serum electrophoresis, reversal of the A/G ratio, and elevated levels of serum kappa free light chain, kappa:lambda ratio (>100), beta-2 microglobulin, and serum IgG. Serum lambda free light chain, IgM, and IgA levels were within normal limits (Figure 9, Table 2). Collectively, these findings were consistent with a diagnosis of PCM, IgG type, with kappa light chain restriction and aberrant CD4 positivity.

**Figure 9:**
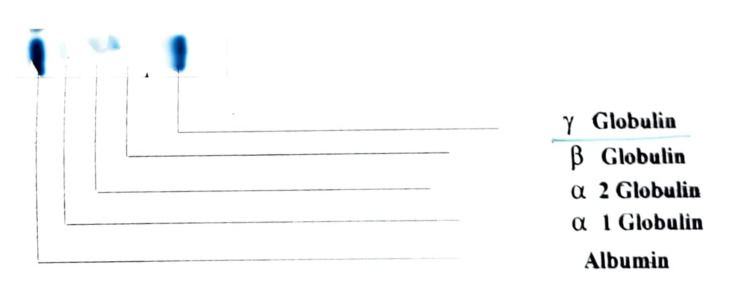
Serum protein electrophoresis Dense band in the gamma globulin region (M band) with hypoalbuminemia, suggestive of monoclonal gammopathy.

The patient was advised chemotherapy with the cyclophosphamide, bortezomib, and dexamethasone (CyBorD) regimen but opted out due to financial constraints. The patient is currently undergoing conservative symptomatic management and packed cell transfusions at a local hospital.

## Discussion

PCM is a clonal proliferation of malignant plasma cells characterized by bone marrow infiltration and end-organ damage, which includes hypercalcemia, renal impairment, anemia, and bone lesions. While the disease most commonly presents with multifocal skeletal involvement, localized plasmacytomas may represent the initial manifestation, either as solitary lesions or as part of systemic disease. Solitary plasmacytomas account for approximately 2-5% of PCNs and carry a substantial risk of progression to overt myeloma, reported in up to 50-80% of cases over time [1]. With osseous forms predominating in the axial skeleton (vertebrae and pelvis), solitary plasmacytomas usually present as painful, lytic bone lesions or soft-tissue masses. Shoulder involvement is extremely uncommon, accounting for less than 5% of solitary bone plasmacytomas [1].

Extramedullary plasmacytoma accounts for 3% of PCNs and less than 1% of all head and neck malignancies [7]. Within this subset, the sinonasal tract and oropharynx are the most frequently involved sites, whereas tongue involvement is distinctly rare, accounting for less than 1% of cases. The present case is notable for simultaneous involvement of the tongue and shoulder, an unusual dual-site presentation at diagnosis that raises the possibility of early disseminated disease with extramedullary tropism. Such presentations are clinically significant, as extramedullary disease in PCM has been associated with aggressive behavior and inferior outcomes. A 2021 case report described an 80-year-old with a dorsal tongue lesion confirmed as EMP with negative marrow involvement. Tongue swelling in PCM may also stem from amyloidosis, where light-chain deposition causes macroglossia with firm, rubbery enlargement and indentations, as reported in 10-15% of primary amyloidosis-associated myeloma [7].

PCNs exhibit diverse morphologies that frequently mimic other hematopoietic and non-hematopoietic malignancies [8]. Over the years, authors have studied and described numerous morphological subtypes of PCNs, some even with their clinical implications. A few of them include lymphoid type, IgA type, pleomorphic, small cell type, cleaved, polymorphous, and blastic type [9,10]. These variants frequently overlap with both hematopoietic and non-hematopoietic malignancies, creating substantial diagnostic challenges. While small-cell and cleaved-cell variants may be mistaken for low-grade B-cell lymphomas, monocytoid, pronormoblast-like, megakaryocytoid, blastoid, and spindle cell forms can mimic hematopoietic neoplasms such as leukemia, myelodysplastic syndromes, and myeloproliferative neoplasms [11]. Furthermore, PCNs with non-hematopoietic morphologies, like signet ring, spindle, anaplastic, and oncocytic variants, may be misdiagnosed as carcinoma, sarcoma, or melanoma [8]. In the present case, the predominance of small- to medium-sized cells with a diffuse growth pattern and high proliferative activity initially suggested a neuroendocrine or lymphoproliferative neoplasm. This diagnostic ambiguity, well recognized in the literature, underscores the need for a broad differential when evaluating poorly differentiated extranodal tumors.

Beyond morphological heterogeneity, PCNs may demonstrate unexpected immunophenotypic divergence. In this case, it was aberrant CD4 positivity that significantly complicated the diagnostic evaluation. Lineage infidelity is uncommon in terminally differentiated B-cell lymphomas, although cases of aberrant expression of T-cell markers like CD2, CD3, CD4, CD5, CD7, or CD8 have been rarely reported in various non-Hodgkin lymphomas [12]. Similar to other hematologic neoplasms, PCNs may exhibit aberrant expression of antigens from different lineages, most commonly involving B-cell or myeloid markers (e.g., CD10 or TdT), whereas expression of T-cell antigens is distinctly uncommon [12,13].

A comprehensive review by Soliman et al. analyzing 22 cases of PCNs with aberrant T-cell marker expression demonstrated positivity of CD3 in 68%, CD4 in 50%, CD7 in 13.6%, CD2 in 9%, and CD8 in 4.5%; all of them were associated with dismal prognosis and short survival time. Notably, a significant proportion of the patients (10/16) presented as solitary extramedullary plasmacytoma, most commonly affecting the skin/soft tissue and lymph nodes [12]. This paralleled the extramedullary manifestations observed in the current case. A study by Hao et al. evaluating 19 newly diagnosed cases of Waldenström’s macroglobulinemia identified acquisition of T-cell antigens (CD3 and CD8) in five cases, confirmed by gene expression profiling, flow cytometry, and immunofluorescence [14]. Additionally, sporadic reports have documented CD4 and CD3 expression in PCM with plasmablastic differentiation [5,6,15].

Proposed theories to explain this abnormal T-cell expression include sensitivity and specificity of the T-cell antibody itself, downregulation of B-cell transcriptional factors, transdifferentiation of neoplastic cells, and Epstein-Barr virus infection [16]. Terminal differentiation of B cells into plasma cells is regulated by a coordinated interplay between PAX5, which preserves B-cell identity, and BLIMP1 (PRDM1), which suppresses the B-cell program and promotes plasma cell maturation. Previous animal experiments have demonstrated that mature B cells may be plastic rather than strictly committed to the B-cell fate. Dedifferentiation of mature B cells in peripheral lymphoid tissues following loss of PAX5 allows these cells to turn in vivo back into progenitors, which are capable of differentiating into T cells that not only carry immunoglobulin gene rearrangements but also express T-cell antigens and function as T cells [17].

Gorczyca demonstrated that immunophenotype is primarily abnormal in poorly differentiated or anaplastic myelomas, which are linked to a poor prognosis [18]. In one study, T-cell antigen expression was reported in six patients with PCM, five of whom had relapsed disease, and was associated with markedly short survival, indicating a potential association with adverse prognosis [13]. The high Ki-67 index observed in the present case (~90%) further supports an aggressive biological phenotype. Additionally, two cases of relapsed PCNs with CD3 expression have been reported [6,19].

Importantly, aberrant CD4 expression may lead to significant diagnostic pitfalls, particularly in cases with undifferentiated morphology. As highlighted by Pan et al., such cases may be misclassified as peripheral T-cell lymphomas, potentially resulting in inappropriate therapeutic strategies [12,15]. This underscores the critical importance of employing a comprehensive immunohistochemical panel even in cases with atypical morphology or unexpected antigen expression.

This case underscores the value of close clinicopathological correlation in achieving an accurate diagnosis. Correlating histomorphology with immunophenotyping, imaging, and laboratory findings was key to confirming PCM. Such an integrated approach is especially important in atypical presentations, where a single modality may be misleading.

Overall, the present case highlights two critical diagnostic challenges in PCNs: (1) significant morphological variability that may mimic other malignancies and (2) rare but clinically relevant immunophenotypic aberrancies such as CD4 expression. Awareness of these features is essential to avoid misdiagnosis, particularly in extramedullary and poorly differentiated presentations.

## Conclusions

If the typical histologic appearance is evident, the diagnosis of PCM is usually straightforward. However, in the absence of conventional clinical features, diagnosis may be considerably more challenging, increasing the risk of misdiagnosis. In such circumstances, a broad immunohistochemical panel including multiple B-cell and T-cell markers, along with CD138, kappa, and lambda light chains, is essential to exclude a PCN. Correlation of morphologic and immunophenotypic findings remains critical for accurate diagnosis. Pathologists should also be aware that lineage infidelity may occasionally occur in poorly differentiated hematologic neoplasms and should be considered during diagnostic evaluation.

## References

[1] (2022). WHO Classification of Tumours, 5th Edition. 5th Edition, Volume 11: Hematolymphoid Tumours. Lyon (France).

[2] Bergstrom DJ, Kotb R, Louzada ML, Sutherland HJ, Tavoularis S, Venner CP (2020). Consensus guidelines on the diagnosis of multiple myeloma and related disorders: recommendations of the Myeloma Canada Research Network Consensus Guideline Consortium. Clin Lymphoma Myeloma Leuk.

[3] Alani A, Choudhuri J, Malonis RJ (2023). Plasma cell neoplasms with unusual anatomic locations, morphology, phenotype, and review of literature: a case series. J Clin Transl Pathol.

[4] Lorsbach RB, Hsi ED, Dogan A, Fend F (2011). Plasma cell myeloma and related neoplasms. Am J Clin Pathol.

[5] Shiroshita K, Kohashi S, Sakamoto M, Kudo H, Nakanishi K, Toyama T (2024). Aberrant CD4 expression in plasmablastic transformation of multiple myeloma. EJHaem.

[6] Mishra P, Kakri S, Gujral S (2017). Plasmablastic transformation of plasma cell myeloma with heterotropic expression of CD3 and CD4: a case report. Acta Clin Belg.

[7] Berdica L, Bushati T, Aga A (2021). Extramedullary plasmacytoma of the tongue: a case report. J Clin Images Med Case Rep.

[8] El Hussein S, Medeiros LJ, Hu S, Lin P, Wang W (2022). The many faces of plasma cell neoplasms: morphological and immunophenotypical variants of the great imitator. Pathology.

[9] Banerjee SS, Verma S, Shanks JH (2004). Morphological variants of plasma cell tumours. Histopathology.

[10] Zukerberg LR, Ferry JA, Conlon M, Harris NL (1990). Plasma cell myeloma with cleaved, multilobated, and monocytoid nuclei. Am J Clin Pathol.

[11] Mahajan V, Vishwaroop S, Khodaiji S (2023). Unusual plasma cell morphology: a diagnostic dilemma. Indian J Hematol Blood Transfus.

[12] Soliman DS, El Sabah H, Ganwo I (2021). Aberrant acquisition of T-cell associated markers in plasma cell neoplasms: an aggressive disease with extramedullary involvement and very short survival. Mediterr J Hematol Infect Dis.

[13] Spier CM, Grogan TM, Durie BG, Richter LC, Vela E, Frutiger Y, Rangel CS (1990). T-cell antigen-positive multiple myeloma. Mod Pathol.

[14] Hao M, Barlogie B, Tricot G, Liu L, Qiu L, Shaughnessy JD Jr, Zhan F (2019). Gene expression profiling reveals aberrant T-cell marker expression on tumor cells of Waldenström's macroglobulinemia. Clin Cancer Res.

[15] Pan Z, Chen M, Zhang Q (2018). CD3-positive plasmablastic B-cell neoplasms: a diagnostic pitfall. Mod Pathol.

[16] Quach P, Lack M, Ash RM, Cunningham MT, Farrell D (2024). Relapsed multiple myeloma in the gastrointestinal tract with aberrant expression of CD3: a case report. Cureus.

[17] Luo X, Kuklani R, Bains A (2016). Dual CD3 and CD4 positive plasma cell neoplasm with indistinct morphology: a diagnostic pitfall. Pathology.

[18] Gorczyca W (2022). Plasma cell neoplasms. Atlas of Differential Diagnosis in Neoplastic Hematopathology.

[19] Yağcí M, Sucak GT, Akyol G, Haznedar R (2002). Hepatic failure due to CD3+ plasma cell infiltration of the liver in multiple myeloma. Acta Haematol.

